# ZnO Doped Silica Nanoparticles (ZnO@SiO_2_) for Enhanced Electrochemical Detection of Cd^2+^ Ions in Real Samples

**DOI:** 10.3390/s24134179

**Published:** 2024-06-27

**Authors:** Afef Dhaffouli, Michael Holzinger, Soledad Carinelli, Houcine Barhoumi, Pedro A. Salazar-Carballo

**Affiliations:** 1 Laboratory of Interfaces and Advanced Materials, Faculty of Sciences of Monastir, University of Monastir, Monastir 5000, Tunisia; dhaffouliafef@gmail.com (A.D.); houcine.barhoumi@fsm.rnu.tn (H.B.); 2 Department of Chemistry, Faculty of Sciences of Gafsa, University of Gafsa, Gafsa 2100, Tunisia; 3 Department of Molecular Chemistry UMR CNRS 5250, Grenoble-Alpes University, CEDEX 9, 38058 Grenoble, France; michael.holzinger@univ-grenoble-alpes.fr; 4 Laboratory of Sensors, Biosensors and Advanced Materials, Faculty of Health Sciences, University of La Laguna, Campus de Ofra s/n, 38071 La Laguna, Spain; scarinel@ull.edu.es

**Keywords:** electrochemical sensor, Zinc oxide, Porous silica coating, Cd^2+^ detection, ZnO@SiO_2_ NPs

## Abstract

Pollution by heavy metal ions has a serious impact on human health and the environment, which is why the monitoring of heavy metal ions is of great practical importance. In this work, we describe the development of an electrochemical sensor for the detection of cadmium (Cd^2+^) involving the doping of porous SiO_2_ spheres with ZnO nanoparticles. Zinc oxide is chosen as the central dopant in the composite material to increase the conductivity and thus improve the electrochemical detection of Cd^2+^ ions with the SiO_2_ spheres. The resulting composite is characterized by electrochemical spectroscopic XRD and microscopic methods. As a result, the developed sensor shows good selectivity towards the targeted Cd^2+^ ions compared to other divalent ions. After optimization of the experimental conditions, the electrochemical sensor shows two different linear ranges between 2.5 × 10^−11^ molL^−1^ to 1.75 × 10^−10^ molL^−1^ and 2 × 10^−9^ molL^−1^ to 1.75 × 10^−9^ molL^−1^, indicating a change from diffusion-controlled to surface-controlled oxidation of Cd^2+^. A detection limit was reached at 4.4 × 10^−11^ molL^−1^. In addition, it offers good repeatability and recovery, and can detect accurate trace amounts of Cd^2+^ ions in real samples such as tap water or seawater by spiking these samples with known Cd^2+^ concentrations. This setup also provides satisfactory recovery rates in the range of 89–102%.

## 1. Introduction

Cadmium is present in the environment in elemental form or as various salts that enter drinking water sources through natural processes (leaching from the soil), human activities (product refinement or technological applications), or leaching from certain types of pipes and known components [1]. This not only affects the health of humans, animals, and plants, but also the ability of the environment to sustain life. As heavy metals are not biodegradable, they accumulate in the food chain. According to the World Health Organization (WHO) [2], a heavy metal concentration above the permissible limit can be toxic, carcinogenic, and harmful to human health [3]. Their high water solubility facilitates their spread in the environment and leads to environmental pollution [4]. The maximum level of Cd^2+^ ions in drinking water is 3.0 μgL^−1^, according to US Environmental Protection Agency (USEPA) standards [5]. It accumulates in humans, reacts with enzymes, and generates free radicals [6], leading to serious health problems. Recent studies have classified Cd^2+^ as a type I human carcinogen [7]. Therefore, the reliable detection of Cd^2+^ is of utmost importance. Effective methods for the determination of Cd^2+^ ions require high selectivity and sensitivity [8]. Various methods are used for the detection of heavy metal ions, which are divided into three classes: electrochemical [9,10,11], gravimetric [12,13], and optical detection methods [14].

However, despite the excellent sensitivity and selectivity achieved with these techniques, sophisticated instrumentation and competent personnel for careful sample preparation are unsuitable for on-site screening [15,16,17]. Among the recently developed technologies for Cd^2+^ detection, electrochemical sensors are considered economical because the equipment and operating procedures are generally simpler and less expensive than other technologies [18]. In addition, electrochemical sensors have many advantages, including being cheap, portable (in situ real-time detection), simple, and offering higher sensitivity and specificity than the detection methods already mentioned [19,20,21]. It has been found that that nanocomposites are core components and play an important role in the development of sensitive electrochemical Cd^2+^ sensors. Electrochemical methods are widely used due to their low cost, simple equipment, easy operation, and strong anti-interference capabilities [22,23]. To improve the sensitivity of the electrochemical sensor, various chemically modified electrodes with nanomaterials [24], magnetic substances [25,26], automotive materials [27], and biological composites [28] have been used. However, these methods are limited in their ability to specifically detect Cd^2+^ ions. Although silica nanoparticles (NP) are an electronic insulator, they are widely used in electrochemical analysis due to their large effective surface area with high selectivity and good sensitivity to heavy metal ions [29,30,31,32].

In this work, a sensitive and selective electrochemical sensor based on ZnO@SiO_2_ was fabricated for the detection of Cd^2+^ ions in real samples. First, these particles were prepared by a modified sol-gel method in the presence of tetraethyl orthosilicate (TEOS). In this technique, a layer of silicon dioxide spheres is formed around the ZnO particles at a controlled temperature of 40 °C. The electrochemical performance of the fabricated sensors was investigated under optimized experimental conditions. The results showed that these sensors were very sensitive to Cd^2+^ ions. Furthermore, by comparing the response current of the electrochemical sensor with other metal ions using differential pulse voltammetry (DPV), it was proven that the sensor exhibited increased signal performance for Cd^2+^ ions. The sensor was also tested on real samples, with deviations ranging from 89 to 102% regarding the spiked Cd^2+^ concentration as 100%.

## 2. Materials and Methods

### 2.1. Materials and Reagents

Zinc oxide nanoparticles (ZnO NPs, nanopowder with a particle size < 100 nm), tetraethyl orthosilicate (TEOS), and ethanol (EtOH) were sourced from Sigma-Aldrich. Sodium hydroxide (NaOH), potassium hexacyanoferrate (II) trihydrate (K_4_[Fe(CN)_6_]·3H_2_O), potassium hexacyanoferrate (III) (K_3_[Fe(CN)_6_]), potassium chloride (KCl), copper (II) dichloride (CuCl_2_), lead nitrate (Pb(NO_3_)_2_), cadmium nitrate (Cd(NO_3_)_2_·4H_2_O), nickel (II) dichloride hexahydrate (NiCl_2_·6H_2_O), manganese (II) dichloride tetrahydrate (MnCl_2_·4H_2_O), and iron (II) dichloride (FeCl_2_) were acquired from Sigma-Aldrich through Chimisi and Chimie Tunisie companies (Tunisia). Acetate buffer solution (ABS, 0.1 molL^−1^; pH 5) was formulated by mixing a 0.1 molL^−1^ acetic acid solution (CH_3_COOH, HAc) and a 0.1 molL^−1^ sodium acetate solution (CH_3_COONa; NaAc) in a 1:1 ratio. This ABS served as the supporting electrolyte, with pH adjustment achieved by incorporating solutions of NaOH or HCl.

Varied concentrations of copper (II), lead (II), nickel (II), manganese (II), iron (II), and cadmium (II) were prepared in an acetate buffer solution (ABS) using distillated water. A stock solution of Cd^2^⁺was diluted until the desired trace concentrations were obtained. For each dilution, the solutions were stirred until stabilization of the corresponding concentration. The experiments for the detection of Cd^2+^ concentrations were performed using firstly the lowest concentration followed by the higher concentration.

### 2.2. Preparation of ZnO@SiO2

**Figure 1:**
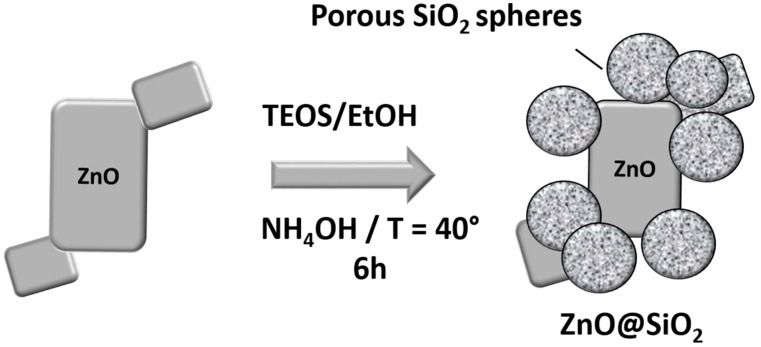
Schematic illustration of the formation of the ZnO@SiO_2_ nanocomposite.

In this step, 0.5 g ZnO nanoparticles were dispersed in 50 mL ethanol and 10 mL water for 30 min in an ultrasonic bath. Then 1.6 mL of ammonia solution (1 molL^−1^) and 8 mL of TEOS were successively added to the Zinc oxide dispersion with vigorous stirring for 6 h at 40 °C. The mixture was washed three times with ethanol and distilled water and finally dried in an oven at 60 °C for 12 h (Figure 1).

### 2.3. Fabrication of ZnO@SiO_2_/GCE Modified Electrodes

Before each use, glassy carbon electrodes (GCE, Ø = 3 mm) were polished with 0.05 μm aluminum oxide powder using emery paper, rinsed with distilled water, and then ultrasonically cleaned in ethanol for 5 min to obtain a reproducible condition. After cleaning the electrode, 1 mg of ZnO@SiO_2_ was dispersed in 1 mL of ethanol by sonication for 30 min and then 3 to 20 μL of the suspension was applied to the GCE. The modified electrodes were then dried at room temperature before being used for measurements.

### 2.4. Characterization of As-Prepared Samples

The morphologies and microstructures of the samples were analyzed by scanning electron microscopy (SEM) using a JEOL JEM-2100 FX microscope (JEOL Ltd., Tokyo, Japan). Transmission electron microscopy (TEM) images were obtained using a JEOL 2100 microscope at 200 kV (JEOL Ltd., Tokyo, Japan). X-ray diffraction patterns for powder samples were obtained with a Philips PanalyticalX’Pert powder diffractometer using CuKα (λ = 1540 Å) radiation in the 2θ range from 5° to 80°. Infrared (IR) spectra were recorded using a PerkinElmer Spectrum IR version 10.6.2 spectrometer and ultraviolet-visible spectroscopy was performed using a Beckman DU640 UV/Vis to further characterize the samples. Electrochemical measurements were performed with a computer-controlled Au-tolab PG Potentiostat/Galvanostat (AUT 83965) and controlled by Autolab’s NOVA 2.1.6 software (Metrohm, Switzerland). The measurements were performed in a traditional three-electrode cell configuration. The working electrode consisted of a modified GCE, while a platinum wire served as the counter electrode and Ag/AgCl (3 molL^−1^ KCl) as the reference electrode. In brief, Cd^2+^ ions were detected using differential pulse voltammetry (DPV). For this purpose, the Cd^2+^ ions were first reduced and accumulated on the sensor surface at −1 V vs. Ag/AgCl for 5 min. Subsequently, a potential sweep from −1 to −0.4 V was applied to detect the target species under optimized conditions (see Supplementary Data).

## 3. Results

### 3.1. Material Characterization

#### 3.1.1. Morphology and Structure of ZnO@SiO_2_

**Figure 2:**
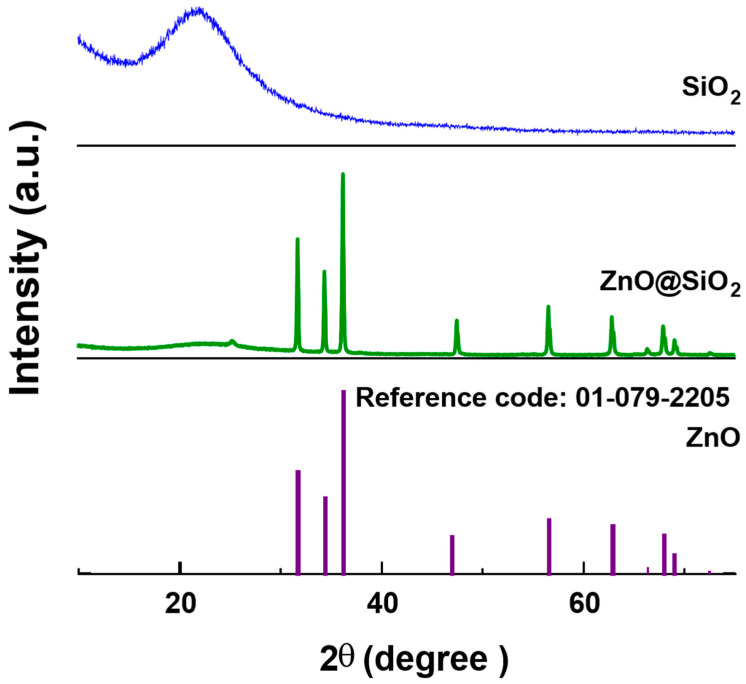
XRD patterns of SiO_2_, ZnO@SiO_2,_ and the reference pattern of ZnO (01-079-2205).

The chemical nature and phase composition of the ZnO@SiO_2_ nanocomposite were analyzed using the XRD technique. The main peaks in the experimental diffractogram are shown in Figure 2. The characteristic peaks at 2θ = 31.76°, 34.41°, 36.25°, 47.53°, 56.59°, 62.85°, 66.37°, 67.94°, 69.08°, and 76.95° observed in the XRD diffractogram were assigned to planes (100), (002), (101), (102), (110), (103), (200), (112), (201), and (202), respectively. These results confirm the existence of ZnO with a hexagonal wurtzite phase (space group: P63mc, JCPDS Chart No. 01-079-2205). The XRD pattern for SiO_2_ shows a characteristic broad band centered at about 2θ = 23° associated with the amorphous phase of silica. This broad band is due to the phenomenon of diffuse scattering often observed in amorphous materials. The diffractogram of the ZnO@SiO_2_ configuration showed that the crystal structure of ZnO was not affected by the presence of SiO_2_ [33]. This can be attributed to the good dispersion of the ZnO nanoparticles in the SiO_2_ matrix [34]. These results are in agreement with our previous studies [35,36].

#### 3.1.2. Electron Microscopy Analysis

**Figure 3:**
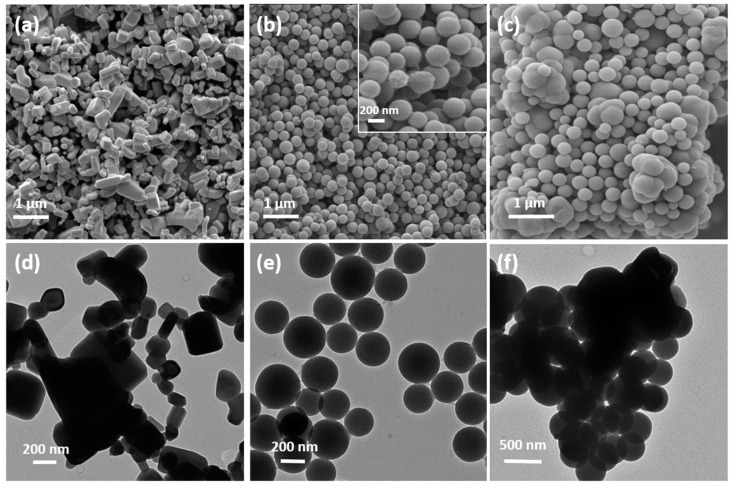
SEM image of (**a**) commercial ZnO NPs, (**b**) synthesized SiO_2_ NPs with a zoom inset, and (**c**) ZnO@SiO_2_.agglomerates. Representative TEM images of (**d**) commercial ZnO NPs, (**e**) synthesized SiO_2_ NPs, and (**f**) ZnO@SiO_2_.

In general, the morphology of almost all metal oxide composites (MO@SiO_2_) tends to form agglomerates of nanoparticles [37]. Figure 3a shows the scanning electron microscopy (SEM) images of commercial ZnO NPs. The particles have a cubic shape and a rather large size distribution between 100 nm and several micrometers. Figure 3b shows the synthesized SiO_2_ spheres with an average size of 250 nm. The inset is a magnification of the micrograph to reveal the porous, almost fuzzy, morphology of the NP surface. Figure 3c is a representative SEM image of the ZnO@SiO_2_ composite. The ZnO particles are mainly covered by the SiO_2_ spheres and only a few ZnO particles with a SiO_2_ coating can be seen. Figure 3d–f are TEM images of ZnO particles, SiO_2_ spheres, and the ZnO@SiO_2_ composite, respectively. ZnO consists of agglomerates of particles with a broad size distribution, which explains the need for ultrasound to disperse these particles. The SiO_2_ spheres also form agglomerates, in which the cross-linked SiO_2_ is clearly visible in the TEM image. The ZnO@SiO_2_ composite forms denser clusters of SiO_2_ spheres around the ZnO particle. However, the formation of a SiO_2_ layer on the ZnO particles is not clearly visible in this microscopic study.

#### 3.1.3. Characterization of the Prepared Materials by Infrared Spectroscopy

**Figure 4:**
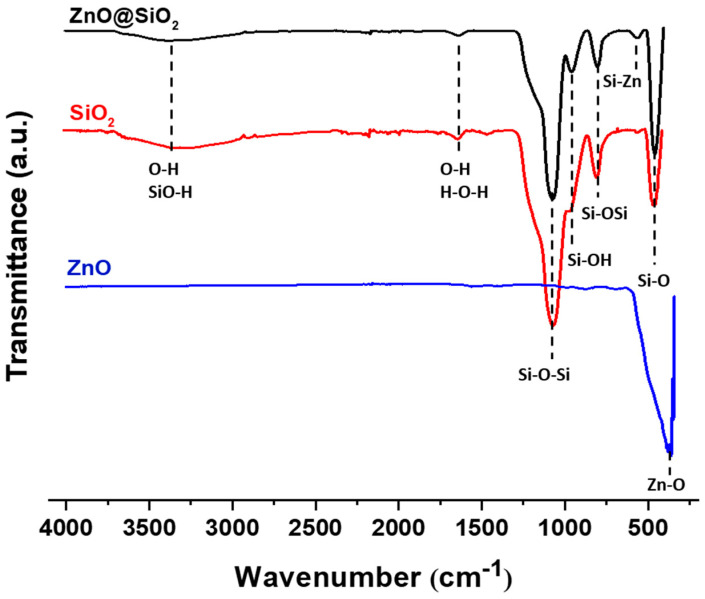
FT-IR spectra of ZnO, SiO_2_, and ZnO@SiO_2_ NPs.

Figure 4 shows the FTIR spectra of ZnO, SiO_2_, and ZnO@SiO_2_ samples. Absorption bands at 451, 794, and 1065 cm^−1^ are observed for the SiO_2_ nanoparticles. These bands are close to the bands described in the literature [38] (466, 808, and 1100 cm^−1^) and are consistent with the SiO_2_ bonding structure. The band corresponds to the symmetric stretching vibration between Si-O bonds of Si-O-Si [39]. Changes in the band characteristics of SiO_2_ indicate that O-Si-O is perturbed by the presence of oxides in its environment. Studies show that in a mixed crystal, the substitution or filling of vacancies can lead to a shift in the fundamental transverse optical modes. The ZnO spectrum shows only one strong absorption peak at 359 cm^−1^. This is the characteristic band of the monoclinic phase of pure Zn-O [40]. The band at 3300 cm^−1^ in the SiO_2_ and ZnO@SiO_2_ spectra indicates the presence of water, which is due to moisture absorption from the environment, the -OH stretching vibration of H_2_O, and the -OH stretching vibration of Si-OH. In addition to the characteristic peaks of ZnO and SiO_2_, the spectrum of ZnO@SiO_2_ samples exhibits another small absorption peak at 556 cm^−1^, which is due to the formation of some Si-O-Zn bonds. These results are in agreement with the results of FTIR studies in the literature [41,42].

#### 3.1.4. UV–Vis Spectra

**Figure 5:**
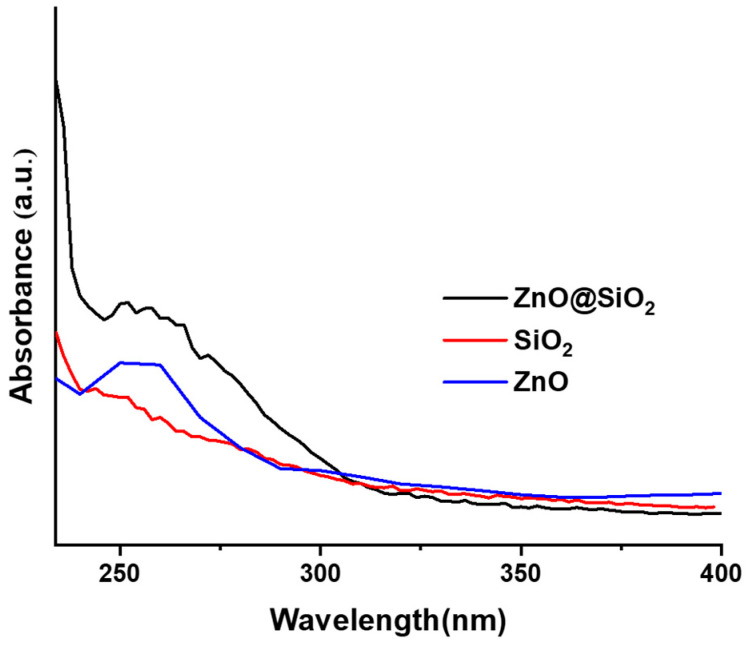
UV–Vis spectra for samples of SiO_2_ and ZnO@SiO_2_.

Figure 5 shows the UV-Vis absorption spectra of ZnO, SiO_2_, and ZnO@SiO_2_, measured in the wavelength range between 228 nm and 400 nm. In contrast to the spectra of ZnO@SiO_2_ and ZnO, the spectrum of the pure SiO_2_ spheres does not show a clearly resolved absorption band, which confirms that the band found in the spectrum of ZnO@SiO_2_ corresponds to the ZnO in the composite. The spectrum of ZnO@SiO_2_ shows a clear band between 240 nm and 300 nm, while the band of ZnO@SiO_2_ appears to be broader, indicating an increase in the transition levels due to the SiO_2_ spheres bound to the ZnO [43]. In a similar study on a CaO@SiO_2_ nanocomposite, the maximum absorption wavelength was reported to be 300 nm [44], which is consistent with the optical properties of the present study. The narrow bandwidth at 270 nm is the result of small ZnO nanoparticles remaining in the porous silica structure [38,45].

### 3.2. Electrochemical Studies

#### 3.2.1. Optimization of Experimental Variables

To evaluate the effect of different parameters for the detection of Cd^2+^ with ZnO@SiO_2_ modified electrodes, we determined the optimal conditions, such as the amount of composite, drying time, pH, and accumulation time. As for the amount, different volumes (3 μL to 20 μL) of ZnO@SiO_2_ solution (1 mg mL^−1^ in ethanol) were applied to the GCE surface, and the highest DPV signal was obtained at 7 µL (Figure S1a). Higher amounts obviously passivate the electrode, which is due to the insulating character of SiO_2_, despite the ZnO doping. We also observed that the drying time of the composite significantly affects the sensor performance. At a drying time of 2 h, the response current reaches its maximum, and at more than 2 h, the response current decreases significantly (Figure S1b). This decrease in current can be explained by the fact that our ZnO@SiO_2_ layer requires a certain amount of moisture to interact sufficiently with the Cd^2+^ ions. If it is too dry, the ZnO@SiO_2_ becomes hydrophobic and the pores are not properly filled by the analyte solutions. To investigate the effects of pH on the response of the electrode, DPV experiments were performed in the pH range from 3 to 8, as shown in Figure S1c. An increase in pH from 3.0 to 5.0 leads to an increase in peak current at −0.78 V vs. Ag/AgCl, with the maximum intensity for Cd^2+^ ions being reached at pH 5.0. Subsequently, the peak currents decreased with increasing pH values. At a mildly acidic pH value, Cd^2+^ is completely ionized, while at higher pH values hydroxides are formed which impair the oxidation capacity of the Cd^2+^ ions. Conversely, at lower pH values (approx. < 4) protons can compete with Cd^2+^ ions for the binding sites.

**Figure 6:**
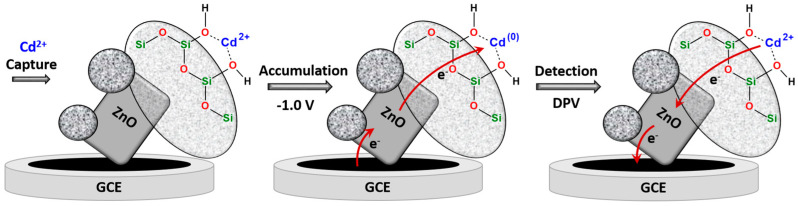
Schematic representation of the adsorption of Cd^2+^ ion of the SiO_2_ surface via polar interactions, the formation of Cd^(0)^ during accumulation via ZnO mediated electron transfers, and reformation of Cd^2+^ during DPV measurements.

Finally, the effect of incubation time on peak current intensity was investigated between 1 and 10 min in a 0.1 molL^−1^ acetate buffer solution at pH 5.0 containing 10^−9^ molL^−1^ Cd^2+^. Figure S1d shows that the current increases with increasing accumulation time from 1 min to 5 min, which can be attributed to the significant increase in the oxidation of the metal ions on the ZnO@SiO_2_/GCE surface. These results confirm that the ZnO@SiO_2_ nanoparticles have a good ability to rapidly accumulate the analyte due to their large active surface area. After 5 min at −1 V vs. Ag/AgCl, the intensity of the current peak decreased. The accumulation time and potential serves to reduce the captured Cd^2+^ ions to Cd^0^, which are then re-oxidized to Cd^2+^ during the DPV measurements. Longer accumulation times had no positive effect on the response, indicating that the electrode surface had reached saturation of the Cd^2+^ that can be reduced and oxidized. Figure 6 illustrates the capture and adsorption of Cd^2+^ ions on the surface bound silanol (Si-OH) functions, most likely via dominant polar interactions because at pH 5 these functions can be considered as completely protonated. These ions are reduced to Cd^(0)^ during accumulation and concentration at −1.0 V vs. Ag/AgCl. It is further shown that the pathways of electrons are mediated via the ZnO particles to the SiO_2_ shell. During reverse potential scanning using DPV, Cd^(0)^ is re-oxidized to Cd^2+^, giving the electrochemical signal.

#### 3.2.2. Detection of Cd^2+^

**Figure 7:**
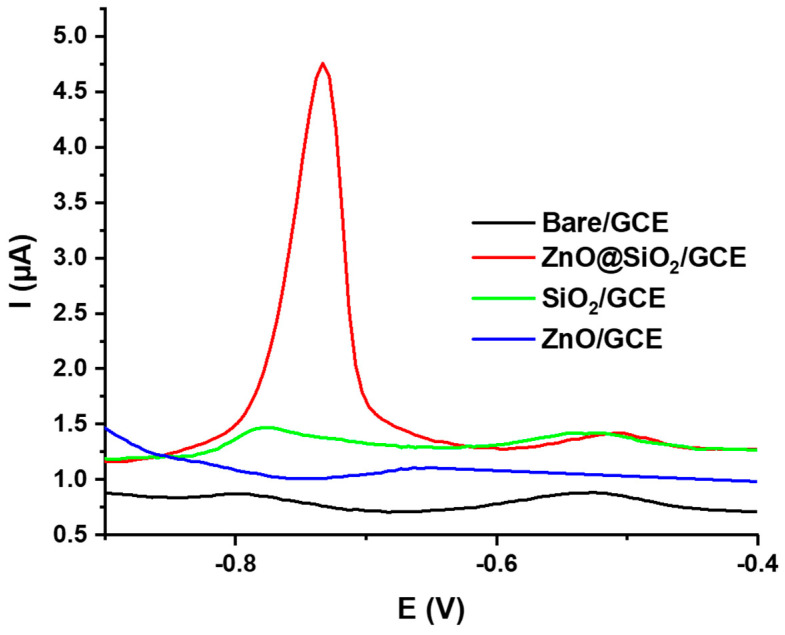
DPV results of bare GCE, SiO_2_/GCE, ZnO/GCE, and ZnO@SiO_2_/GCE electrodes measured in 1.75 × 10^−9^ molL^−1^ Cd^2+^. Data were recorded after drop casting 7 µL of a 1 mg mL^−1^ ZnO@SiO_2_ solution in ethanol, a drying time of 2 h, and an accumulation time of 5 min in 0.1 molL^−1^ in ABS buffer at pH 5.

After optimizing the experimental parameters, we investigated the ability of the ZnO@SiO_2_-modified GCE as an electrochemical sensor for the detection of Cd^2+^ ions in aqueous solutions. The DPVs of a bare GCE, SiO_2_/GCE, and ZnO@SiO_2_/GCE electrodes were recorded in a 0.1 molL^−1^ ABS (pH = 5:0) solution containing 1.75 × 10^−9^ molL^−1^ Cd^2+^ (Figure 7). No obvious response was observed for the bare GCE and ZnO-modified electrode when the accumulation time was 5 min. A small peak with an oxidation current of 1.3 µA was observed for the SiO_2_-modified electrode, which is much lower compared to the ZnO@SiO_2_-modified electrode, which showed a strong reaction peak at −0.78 V vs. Ag/AgCl with a peak current Ipeak = 4.75 μA. This significant difference can be attributed to the insulating nature of SiO_2_, which prevents signal trapping for Cd^2+^ detection. Doping with conductive ZnO particles significantly improves the electron transfer between the electrode and the trapped Cd^2+^ ions.

**Figure 8:**
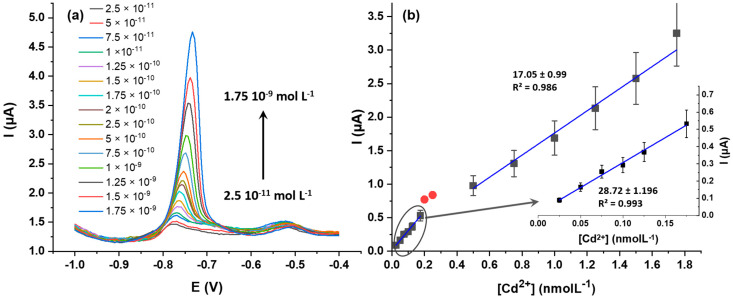
(**a**) Differential pulse voltammograms for Cd^2+^ at varying concentrations using a ZnO@SiO_2_/GCE electrode in 0.1 molL^−1^ ABS buffer pH 5) and (**b**) calibration curves for Cd^2+^ detection on ZnO@SiO_2_/GCE electrodes. The concentration range between 0.2 and 0.5 nmolL^−1^ (red points) are not be considered due to the change in the electrochemical Cd^2+^ oxidation process.

This observation proves the suitability of ZnO@SiO_2_ as a sensitive layer for the electrochemical detection of Cd^2+^ ions. Figure 8a shows the DPV responses of ZnO@SiO_2_/GCE at different Cd_2+_ concentrations. After baseline correction for each DPV, the resulting calibration curve (Figure 8b) shows two linear regions, with linearity values of R^2^ = 0.993 for low concentrations and R^2^ = 0.986 for higher concentrations. These two linearities can be attributed to the change in the electrochemical Cd^2+^ oxidation from diffusion-controlled domain at lower concentrations (from 2.5 × 10^−11^ molL^−1^ to 1.75 × 10^−10^ molL^−1^) and to surface-controlled processes at higher concentrations (from 2 × 10^−10^ molL^−1^ to 1.75 × 10^−9^ molL^−1^). The linearity is not perfect, but satisfying considering the overall low concentration range. At higher concentrations, the electrode surface becomes saturated where no current increase could be measured above 10^−6^ molL^−1^. The detection limit is remarkably low, reaching 2.5 × 10^–11^ mol L^−1^ (S/N = 3). 

This sensor setup was also compared with SiO_2_/GCE without ZnO dopants (Figure S2). A consistent DPV response could be obtained at much higher concentrations with a linear range between 5 × 10^−7^ and 2.5 × 10^−6^ molL^−1^. ZnO doping thus enables monitoring of trace amounts of Cd^2+^ ions, while SiO_2_ spheres alone are better suited for quantification of Cd^2+^ ions in highly polluted environments. By focusing on the detection of trace amounts, the advantageous properties of ZnO doping in this composite can be compared to recently reported sensors [46,47,48,49,50,51,52,53,54,55,56], as summarized in Table 1.

#### 3.2.3. Appropriateness of ZnO@SiO_2_ for Specific Cd^2+^ Detection

**Figure 9:**
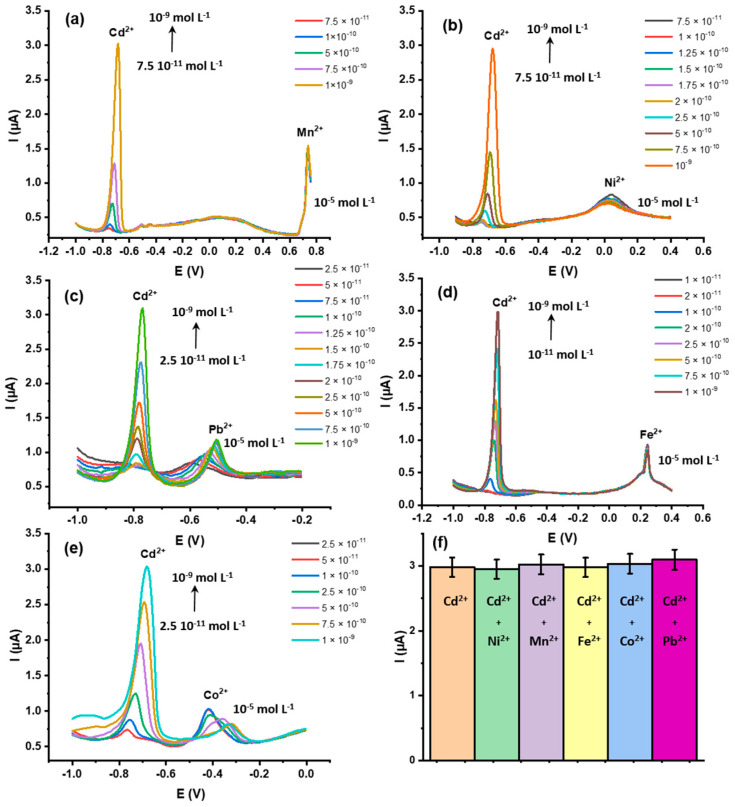
Differential pulse voltammograms of ZnO@SiO_2_/GCE with different Cd^2+^ concentrations in the presence of potential interfering ions (**a**) Mn^2+^, (**b**) Ni^2+^, (**c**) Pb^2+^, (**d**) Fe^2+^, and (**e**) Co^2+^). (**f**) Histogram of the maximum current peaks for Cd^2+^ at 10^−9^ molL^−1^ in the presence of the different M^2+^ ions (10^−5^ molL^−1^). The electrodes were prepared by drop casting 7 µL of a 1 mg mL^−1^ ZnO@SiO_2_ solution in ethanol, with a drying time of 2 h, and the measurements were taken with 5 min of accumulation time in 0.1 mol L^−1^ ABS buffer at pH 5.

The ZnO@SiO_2_ nanocomposite was selected for the specific detection of Cd^2+^ because it has a cavity structure with suitable sizes for the investigated model ion. To evaluate the sensor efficiency, ionic species with redox potentials close to Cd^2+^, such as Ni^2+^, Mn^2+^, Fe^2+^, Co^2+^, and Pb^2+^, were selected and used as interfering ions. As can be seen in Figure 9, the ZnO@SiO_2_ electrode showed a significant response current for Cd^2+^ ions at −0.78 V versus Ag/AgCl and significantly lower currents for the other divalent ions within this potential range. Only Mn^2+^ showed a good response, but the potential (−0.73 V) is clearly separated from the Cd^2+^ peak potential. In addition, a higher concentration (10^−5^ molL^−1^) was used for all tested ions than for Cd^2+^ (max. 1 × 10^−9^). We further investigated possible changes in the peak currents with Cd^2+^ at different concentrations in the presence of these potentially interfering ions (Figure 9a–e). Within the concentration range between 1 × 10^−9^ and 1 × 10^−11^ molL^−1^, the signals are well reproducible, making this setup suitable for the detection of trace Cd^2+^ in contaminated samples [57,58]. The histogram in Figure 9f confirms the good reproducibility of the sensor formation (*n* = 3).

#### 3.2.4. Repeatability and Recovery

**Figure 10:**
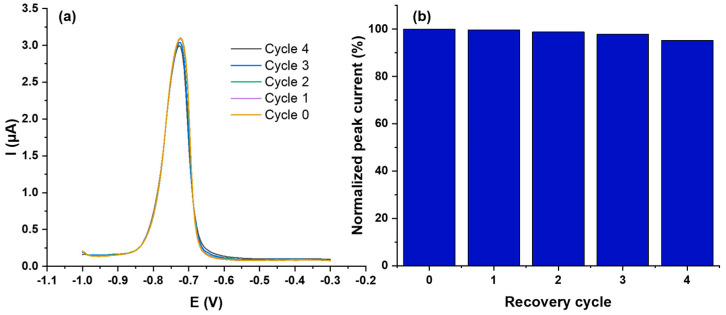
(**a**) DPV Sensor responses to 10^−5^ molL^−1^ of Cd^2+^ ions using one prepared ZnO@SiO_2_/GCE modified electrode five times with four recovery cycles. (**b**) The resulting histogram visualizing the deviations. The measurements were taken in 1 molL^−1^ EDTA in 0.1 molL^−1^ ABS buffer at pH 5.

Repeatability and stability are crucial for the reliable performance of electrochemical sensors. Therefore, we investigated these aspects with respect to the proposed sensor. To evaluate the repeatability of ZnO@SiO_2_/GCE electrodes, six different electrodes (Figure S3) coated with the same amount of the composite material were formed and subjected to DPV analysis at 10^−9^ molL^−1^ Cd^2+^ in 0.1 molL^−1^ acetate buffer (pH 5). The oxidation peak potential for Cd^2+^ was −0.78 V and the peak current showed a relative standard deviation (RSD) of 6.28%, which is still acceptable considering the low Cd^2+^ concentration (10^−9^ molL^−1^ = 8 × 10^−3^ µgL^−1^) and shows the good reproducibility of the presented protocol. The repeatability of the sensor was also investigated. After Cd^2+^ determination using the DPV method (recovery cycle 0), the tested sensor was immersed in 1 molL^−1^ EDTA solution for 5 min and reconditioned for subsequent Cd^2+^ determinations (*n* = 4). After immersion in an aqueous EDTA solution (1 molL^−1^), the electrode was rinsed three times with distilled water and dried in air at room temperature for 1 h. The efficiency of this cleaning procedure is shown in Figure S4. Figure 10 shows the results of five consecutive recovery cycles using 10^−9^ molL^−1^ Cd^2+^ in a 0.1 molL^−1^ ABS buffer solution (pH 5). The current intensity decreased only slightly after the first cycle and reached 99.7% of the original peak current. The following cycles showed greater performance losses, but after five recovery cycles 95.2% could be achieved compared to the freshly manufactured electrode. These results underline the ability to reuse these electrodes several times.

#### 3.2.5. Applications to Real Samples Analysis

To test the reliability of our ZnO@SiO_2_/GCE sensor, four different water samples were analyzed: tap water from the laboratory, colored water from a local store (laboratory of the textile department), seawater from Skanes, and seawater from Ksibet Elmediouni (Monastir, Tunisia). All water samples were first diluted with ABS tampon solution in a ratio of 1:99 to avoid any possible detrimental effects on the sensor performance by chloride ions. Then the samples were spiked with Cd^2+^ by diluting 1 mL of a Cd^2+^ ABS solution (1.75 × 10^−6^ molL^−1^) with the corresponding volume of the real water sample to obtain the final concentration. The measured peak currents were then included in the generated calibration curve (Figure 8b) to obtain the corresponding concentration. The values obtained correlate well with the amount added, with deviations ranging from 89% (tap water) to 102% (seawater from Ksibet Elmediouni). As almost all optimized parameters (quantity, drying, and accumulation time) were met, with the exception of the pH value, these deviations are mainly due to the different pH values of the various samples (see Table 2). These results support the reliability of the proposed Cd2+ sensor for environmental water samples.

## 4. Conclusions

In this study, high performance detection of Cd^2+^ was achieved using glassy carbon electrodes modified with ZnO@SiO_2_ nanocomposites. DPV analysis showed that the modification of commercial electrodes used in portable devices with this nanocomposite (ZnO@SiO_2_) is feasible for the detection of trace amounts of Cd^2+^. The incorporation of ZnO nanoparticles in the core significantly improved the electrochemical performance of the sensor. The experimental results showed that the electrochemical sensor using DPV transduction has good sensitivity and high selectivity. Under optimal experimental conditions, the ZnO@SiO_2_ sensor exhibits two distinct linear ranges between 2.5 × 10^−11^ to 1.75 × 10^−10^ molL^−1^ and 2 × 10^−10^ to 1.75 ×10^−9^ molL^−1^, which are related to a change from diffusion-controlled to surface-controlled oxidation of Cd^2+^. Ultimately, the ZnO@SiO_2_ composite proved to be effective in detecting Cd^2+^ in environmental samples using DPV analysis, which enables reliable detection of Cd^2+^ in the presence of other metal ions.

## Figures and Tables

**Table 1:** Comparison of our setup with other electrochemical sensors for the determination of Cd^2+^ by DPV.

Modified Electrode	Method	Linear Range (molL^−1^)	Detection Limit (molL^−1^)	Reference
Chitosan-carbon nanotubes/GCE	SWASV	1.3 × 10^−5^–3.9 × 10^−5^	7.1 × 10^−6^	[46]
LAL-AuNPs/GCE	SWASV	3.0 × 10^−7^–1.4 × 10^−6^	3.0 × 10^−7^	[47]
Porous carbon-PdNPs/GCE	DPV	5.0 × 10^−7^–5.5 × 10^−6^	4.1 × 10^−8^	[48]
MIL-53(Fe)/GCE	DPV	1.5 × 10^−7^–4.5 × 10^−7^	1.6 × 10^−8^	[49]
Fe_3_O_4_/RGO/GCE	SWASV	0−8 × 10^−7^	5.6 × 10^−8^	[2]
Nano-PPCPE	DPASV	10^−7^–3 × 10^−6^	7.8 × 10^−8^	[50]
PA/PPY/GO/GCE	DPV	4.4 × 10^−8^–1.3 × 10^−6^	1.9 × 10^−8^	[51]
MSK-NPs@GRCPE	DPASV	5 × 10^−11^–2 × 10^−6^	5.44 × 10^−9^	[52]
NH_2_@GCE	DPV	3 × 10^−7^–1.5 × 10^−5^	2 × 10^−7^	[53]
GCE/ZSM-5/Pt (5.4%)	DPV	10^−7^–7.2 × 10^−6^	1.2 × 10^−9^	[54]
PPh_3_/MWCNTs/IL/CPE	DPASV	1 × 10^−10^–1.5 × 10^−7^	7.4 × 10^−5^	[55]
GCE/SBA-15/ZrO_2_ (30%)	SWASV	10^−7^–5.5 × 10^−6^	3.14 × 10^−7^	[56]
SiO_2_/GCE	DPV	5 × 10^−7^–2.5 × 10^−6^	5 × 10^−7^	This work
ZnO@SiO_2_/GCE	DPV	2.5 × 10^−11^–1.75 × 10^−10^ and 2 × 10^−10^–1.75 × 10^−9^	4.4 × 10^–11^	This work

SWASV: square wave anodic stripping voltammetry; DPASV: differential pulse anodic stripping voltammetry; DPV: differential pulse voltammetry; LAL: laser ablation in liquid; Fe_3_O_4_/RGO/GCE: magnetite-reduced graphene oxide modified glassy carbon electrode; Nano-PPCPE: nanoporous pseudo carbon paste electrode; PA/PPY/GO/GCE: Phytic acid-functionalized polypyrrole–graphene oxide functionalized glassy carbon electrode.

**Table 2:** Detection of Cd^2+^ in different environmental water sources.

Sample	Add (molL^−1^)	Found (molL^−1^)	
Tap water	1.75 × 10^–9^	1.57 × 10^–9^	89.71
Tinted water	1.75 × 10^–9^	1.6119 × 10^–9^	92
Sea water (Skanes)	1.75 × 10^–9^	1.7411 × 10^–9^	99.49
Sea water (Ksibet Elmediouni)	1.5 × 10^–9^ 1.75 × 10^–9^	1.52 × 10^–9^ 1.788 × 10^–9^	100.66 102.2

## Data Availability

All experimental data are available and can be shared on request.

## Supplementary Materials

The following supporting information can be downloaded at: https://www.mdpi.com/article/10.3390/s24134179/s1, Figure S1: Optimization of experimental variables. Figure S2: Detection of Cd^2+^ with SiO_2_ nanospheres. Figure S3: Repeatability studies. Figure S4: recovery of the sensor after EDTA treatment.
