# Supplementary material for: ZnO Doped Silica Nanoparticles (ZnO@SiO2) for Enhanced Electrochemical Detection of Cd2+ Ions in Real Samples
Source: Sensors (Basel). 2024 Jun 27;24(13):4179. doi: 10.3390/s24134179 (PMC11244355; doi:10.3390/s24134179)
Supplement: Supplementary file 1 [file sensors-24-04179-s001.zip › sensors-3058101-supplementary.pdf]

sensors

MDPI

Supplementary Materials

ZnO Doped Silica Nanoparticles (ZnO@SiO2) for Enhanced Electrochemical Detection of Cd^{2+} Ions in Real Samples

Afef Dhaffouli, Michael Holzinger, Soledad Carinelli, Houcine Barhoumi and Pedro A. Salazar-Carballo *

1. Optimization of experimental variables

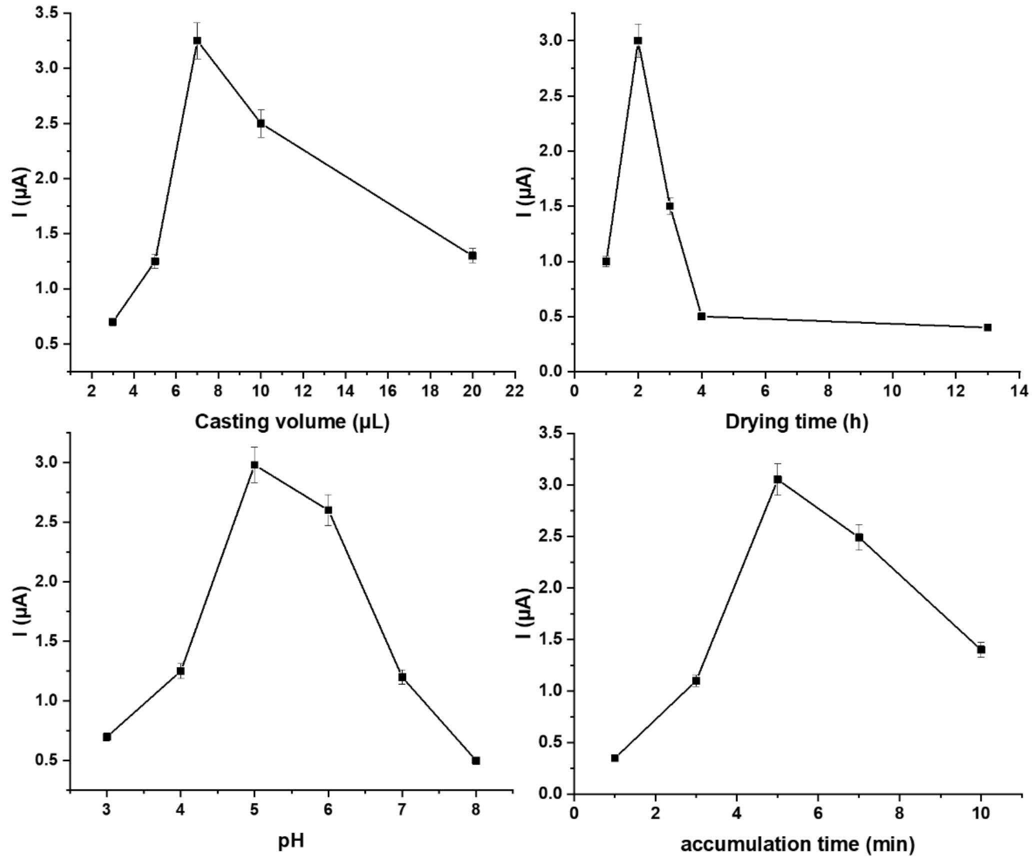

Figure S1. Influence of (a) the amount of matrix deposited on the electrode, (b) Drying time at room temperature, (c) pH values of the buffer solution, and (d) Accumulation time during the detection step, on the voltammetry respnse of $ZnO@SiO_2$/GCE in HAc-NaAc (0.1 mol L^{-1}) contained 10^{-5} mol L^{-1} of Cd^{2+} for optimal DPV signal capture (at -0.78V) under these conditions. (n =3).

2. Detection of Cd^{2+} with SiO_2 nanospheres

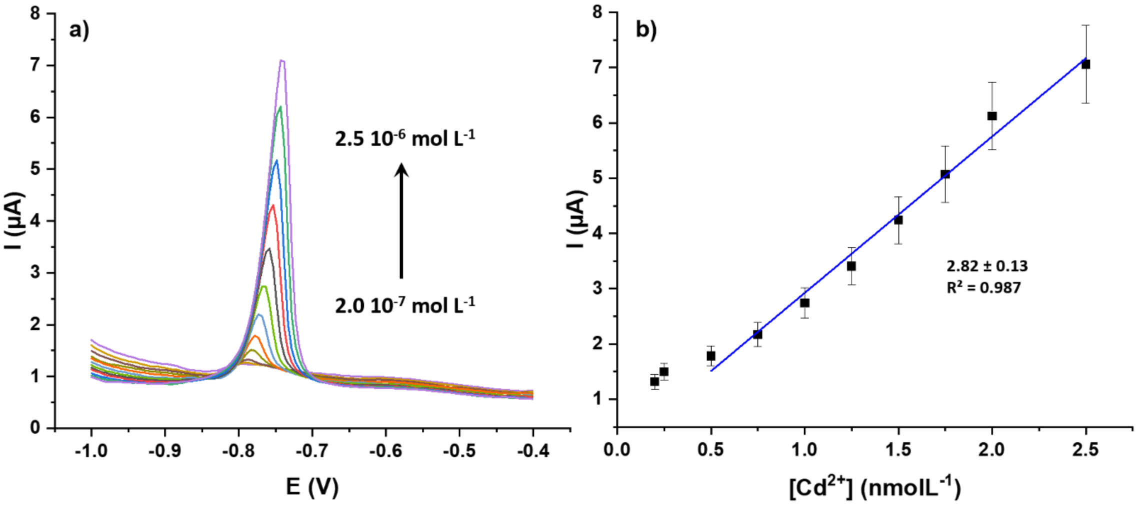

Figure S2. a) Differential pulse voltammograms of Cd^{2+} at varying concentrations with a SiO_2/GCE 0.1 mol L^{-1} ABS buffer pH 5), b) Calibration curves for Cd^{2+} detection on SiO_2/GCE electrode.

3. Repeatability studies

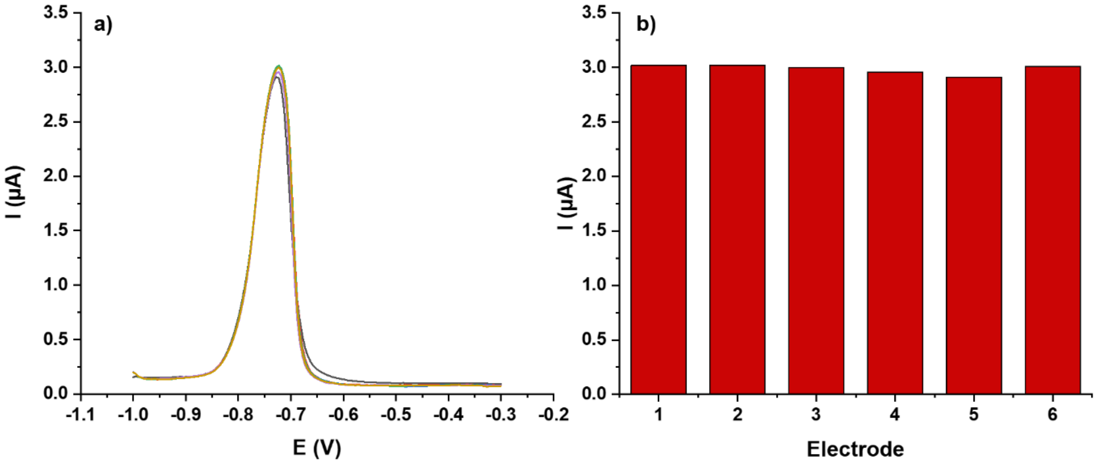

Figure S3. a) Repeatability of the sensor to a concentration of 10^{-9} mol L^{-1} Cd^{2+} using six different ZnO@SiO_2/GCE modified electrodes with b) the resulting histogram visualizing the deviations.

4. Efficiency of EDTA (1 molL^{-1}) cleaning and regeneration of the used electrodes

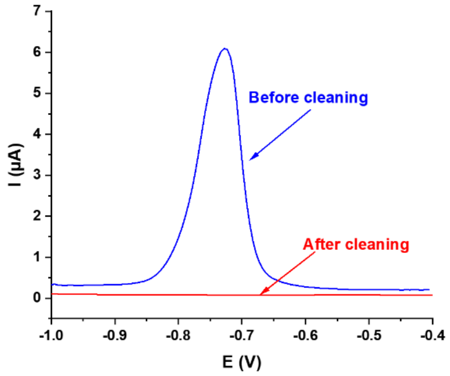

Figure S4. DPV measurements of ZnO@SiO_2/GCE after Cd^{2+} detection and after cleaning using a 1 molL^{-1} aqueous EDTA solution.
